# Characterization of Fibrinogen Binding by Glycoproteins Srr1 and Srr2 of Streptococcus agalactiae*

**DOI:** 10.1074/jbc.M113.513358

**Published:** 2013-10-28

**Authors:** Ho Seong Seo, George Minasov, Ravin Seepersaud, Kelly S. Doran, Ievgeniia Dubrovska, Ludmilla Shuvalova, Wayne F. Anderson, Tina M. Iverson, Paul M. Sullam

**Affiliations:** From the ‡ Division of Infectious Diseases, Veterans Affairs Medical Center, University of California at San Francisco and the Northern California Institute for Research and Education, San Francisco, California 94121,; the § Radiation Biotechnology Research Division, Advanced Radiation Technology Institute, Korea Atomic Energy Research Institute, Jeongeup Si, 580-185, Republic of Korea,; the ¶ Center for Structural Genomics of Infectious Diseases and the Department of Molecular Pharmacology and Biological Chemistry, Northwestern University Feinberg School of Medicine, Chicago, Illinois 60611,; the ‖ Department of Biology and Center for Microbial Sciences, San Diego State University, San Diego, California 92182,; the ** Department of Pediatrics, University of California at San Diego School of Medicine, La Jolla, California 92093, and; the Departments of ‡‡ Pharmacology and; §§ Biochemistry, Vanderbilt University Medical Center, Nashville, Tennessee 37232

**Keywords:** Bacterial Adhesion, Bacterial Pathogenesis, Fibrinogen, Protein Crystallization, Streptococcus, Streptococcus agalactiae, Prot

## Abstract

The serine-rich repeat glycoproteins of Gram-positive bacteria comprise a large family of cell wall proteins. *Streptococcus agalactiae* (group B *streptococcus*, GBS) expresses either Srr1 or Srr2 on its surface, depending on the strain. Srr1 has recently been shown to bind fibrinogen, and this interaction contributes to the pathogenesis of GBS meningitis. Although strains expressing Srr2 appear to be hypervirulent, no ligand for this adhesin has been described. We now demonstrate that Srr2 also binds human fibrinogen and that this interaction promotes GBS attachment to endothelial cells. Recombinant Srr1 and Srr2 bound fibrinogen *in vitro*, with affinities of *K_D_* = 2.1 × 10^−5^ and 3.7 × 10^−6^ m, respectively, as measured by surface plasmon resonance spectroscopy. The binding site for Srr1 and Srr2 was localized to tandem repeats 6–8 of the fibrinogen Aα chain. The structures of both the Srr1 and Srr2 binding regions were determined and, in combination with mutagenesis studies, suggest that both Srr1 and Srr2 interact with a segment of these repeats via a “dock, lock, and latch” mechanism. Moreover, properties of the latch region may account for the increased affinity between Srr2 and fibrinogen. Together, these studies identify how greater affinity of Srr2 for fibrinogen may contribute to the increased virulence associated with Srr2-expressing strains.

## Teaser

**Background:** The serine-rich repeat glycoproteins Srr1 and Srr2 are surface adhesins of *Streptococcus agalactiae* important for pathogenicity.

**Results:** Both Srrs bind tandem repeats of the fibrinogen Aα chain, but Srr2 has greater affinity explained by structure-function analysis of the Srrs.

**Conclusion:** A dock, lock, and latch mechanism describes the Srr-fibrinogen interaction.

**Significance:** The higher affinity of Srr2 may contribute to the hypervirulence of Srr2-expressing strains.

## Introduction

The serine-rich repeat (SRR)^2^ glycoproteins of Gram-positive bacteria are a family of adhesins that are important virulence factors for their respective pathogens (1–3). These bacterial surface components are encoded within large loci that also encode proteins mediating their glycosylation and export. Each SRR protein consists of a long and specialized signal sequence, a short serine-rich region (SRR1), a ligand binding region, a second lengthy SRR region, and a typical LP*X*TG cell wall anchoring motif at the C terminus (4, 5). Although relatively few of the SRR proteins have been studied in detail, the binding regions of the SRR glycoproteins appear to vary significantly in predicted structure and binding properties. Among the best characterized SRR proteins is GspB of *Streptococcus gordonii*, which binds human platelets through its interaction with sialyl-T antigen on the platelet receptor GPIb (6, 7). This interaction appears to be an important event in the pathogenesis of endocarditis, because disruption of GspB binding is associated with a marked reduction in virulence, as tested by animal models of endocardial infection (7, 8). A number of other SRR proteins have been shown to contribute to virulence, including SraP of *Staphylococcus aureus*, PsrP of *Streptococcus pneumoniae*, and UafB of *Staphylococcus saprophyticus* (9–11), although the molecular basis for binding by these other adhesins is somewhat less well defined.

*Streptococcus agalactiae* (group B *Streptococcus,* GBS) is a leading cause of neonatal sepsis, pneumonia, and meningitis (12, 13). In recent decades, this organism has also become a significant cause of invasive infections among adults (14). GBS strains express either one of two SRR proteins, Srr1 or Srr2. Expression of Srr1 by GBS has been shown to contribute to colonization and virulence in models of infection (15–17). Srr1 mediates bacterial binding to cytokeratin 4, which is likely to be important for colonization of the female genital tract and is a risk factor for subsequent invasive disease (17, 18). In addition, we have recently shown that Srr1 binds to human fibrinogen via its interaction with the Aα chain of the protein. Srr1-mediated binding to fibrinogen is important for the attachment of GBS to human brain microvascular endothelial cells (hBMEC), where fibrinogen served as a bridging molecule between Srr1 and the endovascular surface (4).

Sequence comparisons and deletion mutagenesis studies (4) suggest that the interaction between Srr1 and fibrinogen could employ the “dock, lock, and latch” (DLL) mechanism described for several other fibrinogen-binding adhesins, such as ClfB of *S. aureus* and SdrG of *Staphylococcus epidermidis* (19–21). During this binding process, fibrinogen engages a cleft between two IgG-like folds (the N2 and N3 domains) of the binding region. This docking event results in a conformational change of the adhesin, such that the flexible C terminus of the N3 domain (the “latch”) forms a β-strand and completes a β-sheet within the N2 domain, thereby “locking” the ligand in place. Deletion of the latch region of Srr1 is associated with reduced GBS binding *in vitro* to fibrinogen and hBMEC and resulted in attenuated virulence in a mouse model of bacteremia and meningitis (4). These findings indicate that fibrinogen binding via Srr1 may occur via a DLL mechanism and that this interaction enhances pathogenicity.

As compared with Srr1, relatively little is known about the binding properties of Srr2 or its contribution toward GBS virulence. Srr2 has been detected in serotype III strains exclusively and only in isolates belonging to sequence multilocus sequence type 17 (ST-17), a genotype linked epidemiologically to increased invasive disease (16, 22–28). In addition, strains expressing Srr2 were significantly more virulent in a mouse model of neonatal sepsis, as compared with Srr1-expressing strains (16), suggesting that this surface component may at least in part explain the increased virulence associated with ST-17 isolates. ST-17 strains also have higher levels of fibrinogen binding, but the molecular basis for this has not been well defined (28). Delineating the molecular differences between Srr1 and Srr2 could improve our understanding of how Srr2 confers hypervirulence in *S. agalactiae*. We now report that both Srr1 and Srr2 bind to a specific tandem repeat region of fibrinogen Aα chain. Crystal structures and mutagenesis studies indicate that both proteins employ a DLL mechanism for host binding. Moreover, Srr2 has significantly higher binding affinity for fibrinogen as compared with Srr1, and analysis of their structures suggests that the physical positioning of the latch region may underlie this enhanced affinity.

## EXPERIMENTAL PROCEDURES

### 

#### 

##### Reagents

Purified human fibrinogen was obtained from Hematologic Technologies. Rabbit anti-fibrinogen IgG was purchased from Aniara. Rabbit anti-Srr2 IgG was generated by NeoPeptide, using purified recombinant protein corresponding to the binding region (BR) of Srr2.

##### Strains and Growth Conditions

The bacteria and plasmids used in this study are listed in Tables 1 and 2. *S. agalactiae* strains were grown in Todd-Hewitt broth (Difco) supplemented with 0.5% yeast extract (THY). All mutant strains grew comparably well *in vitro*, as compared with parent strains (data not shown). *Escherichia coli* strains DH5α, BL21, and BL21(*DE3*) were grown at 37 °C under aeration in Luria broth (LB; Difco). Antibiotics were added to the media as required.

**TABLE 1:** **Strains**

Strain or plasmid	Genotype or description*^a^*	Source
***E. coli***		
DH5α	F^−^r^−^m^+^Ø80d*lacZ*ΔM15	Invitrogen
BL21 (DE3)	Expression host, inducible T7 RNA polymerase	Novagen

***S. agalactiae***		
COH31	Serotype III	67
PS954	COH31Δ*srr*1, Cm^R^	15
NCTC 10/84	Serotype V, clinical isolate	68
PS2645	NCTC 10/84Δ*srr*1, Cm^R^	15
COH1	Serotype III, clinical isolate, ST-17	69
PS2641	COH1Δ*srr2*, Cm^R^	17, 49
PS2931	PS2641/pDE-Srr1	This study
PS2933	PS2641/pDE-Srr2	This study
H36B	Serotype Ib	70
NCTC 1/82	Serotype IV	4
J48	Serotype III, ST-17	16
NEM316	Serotype III, ST-23	71

*^a^* Erm^R^, erythromycin resistance; Cm^R^, chloramphenicol resistance.

**TABLE 2:** **Plasmids**

Plasmid	Description	Source
pDE123	Streptococcal shuttle vector, Erm^R^	4
pDE123-*srr1*	Vector for expression of Srr1, Erm^R^	4
pDE123-*srr2*	Vector for expression of Srr2, Erm^R^	This study
pET22b(+)	Expression vector, Amp^R^	Novagen
pET28-FLAG	Expression vector with FLAG tag, Kan^R^	32
pET22-Srr1-BR	Vector for expression of Srr1-BR, Amp^R^	4
pET22-Srr2-BR	Vector for expression of Srr2-BR, Amp^R^	This study
pET22-ClfA-BR	Vector for expression of ClfA-BR, Amp^R^	This study
pET28-FLAG-Srr2-BRΔlatch	Vector for expression of FLAG-tagged Srr1 (303–627)	This study
pSET-5S	Streptococcal thermosensitive suicide vector, Cm^R^	72
pSET-5S-*srr2*KO	Vector for deletion of *srr2* gene, Cm^R^	This study
pMAL-C2X	Expression vector with MBP fusion protein	New England Biolabs
pMal-Aα	Vector for expression of MBP-tagged Aα chain	33
pMal-Bβ	Vector for expression of MBP-tagged Bβ chain	33
pMal-γ	Vector for expression of MBP-tagged γ chain	33
pMal-Aα(1–197)	Vector for expression of MBP-tagged Aα variant	4
pMal-Aα(198–610)	Vector for expression of MBP-tagged Aα variant	4
pMal-Aα(198–282)	Vector for expression of MBP-tagged Aα variant	4
pMal-Aα(283–410)	Vector for expression of MBP-tagged Aα variant	4
pMal-Aα(198–282 + 411–610)	Vector for expression of MBP-tagged Aα variant	4
pMal-Aα-RU1–10	Vector for expression of MBP-tagged Aα variant	This study
pMal-Aα-RU1–6	Vector for expression of MBP-tagged Aα variant	This study
pMal-Aα-RU1–7	Vector for expression of MBP-tagged Aα variant	This study
pMal-Aα-RU1–8	Vector for expression of MBP-tagged Aα variant	This study
pMal-Aα-RU1–9	Vector for expression of MBP-tagged Aα variant	This study

##### Cloning and Expression of Srr1-BR and Srr2-BR

Genomic DNA was isolated from GBS NCTC 10/84 and COH1 using Wizard Genomic DNA purification kits (Promega), according to the manufacturer's instructions. PCR products were cloned into pET28-FLAG to express FLAG-tagged versions of Srr1-BR (amino acids 303–641), Srr2-BR (amino acids 303–641), or the latch deletion variant of Srr2-BR (amino acids 303–628). DNA encoding Srr1-BR, Srr2-BR, Srr1-BRΔlatch, Srr2-BRΔlatch, or ClfA-BR (N2N3) were cloned into pET22b(+) (Novagen) or pET28-FLAG. Proteins were purified by either nickel-nitrilotriacetic acid (Promega) or anti-FLAG M2-agarose affinity chromatography (Sigma), according to the manufacturers' instructions.

##### Cloning and Expression of Fibrinogen Chains

DNA of each chain was amplified from cDNA encoding the Aα-, Bβ-, and γ-chains of human fibrinogen and cloned into pMAL-C2X (New England Laboratory) as described previously (29–31). The recombinant proteins were purified by affinity chromatography with amylose resin, according to the manufacturer's instructions (New England Biolabs).

##### Site-directed Mutagenesis

Cysteine replacement mutations were made within latch and latching cleft domains of Srr-BRs by a two-stage PCR procedure. For codon conversion to cysteine in the latching cleft, overlapping primers were used with either primer 3006(NotI)/5003 (N423C) or 3003(N423C)/5006(XhoI) for Srr1-BR and either 3012(NotI)/5009 (N336C) or 3009(N339C)/5012(XhoI) for Srr2-BR to generate overlapping DNA fragments spanning the entire Srr1-BR and Srr2-BR. The two DNA fragments were combined for the second stage PCR and then amplified using primers 3006(NotI)/5006(XhoI, K639C) for the Srr1-BR and 3012(NotI)/5012(XhoI, N541C) for Srr2-BR. Amplified products were digested with the appropriate restriction enzymes and ligated into pET28-FLAG. The constructs were sequenced to confirm that the mutations were correctly positioned and then expressed in *E. coli*, as described above.

##### Construction of Plasmids for Gene Complementation

Genomic DNA was isolated from COH1 and NCTC 10/84 strains, using Wizard Genomic DNA purification kits (Promega). Polymerase chain reaction (PCR) was performed with the primers (Srr1 forward, AAT CTA GAT AGA TTT CTA ATC ACT TAA TTT TAC, and Srr1 reverse, GCT CTA GAA GAA TTC AAA GTA GGT TTA GTC; Srr2 forward, TTT CTA GAT AGC ATT ATT TTT TAA ATA TGG, and Srr2 reverse, TTC TGC AGT TAA TCT TTT TTC TTC TTG C) to amplify *srr1* or *srr2* genes. PCR products were purified, digested, and ligated into pDE123 to express the full-length Srr1 and Srr2.

##### Analysis of Srr2-BR Binding to Fibrinogen by Far Western Blotting

Purified human fibrinogen (0.1 μg) and recombinant fibrinogen truncations (0.5 μg) were separated by electrophoresis in 3–8% NuPAGE Tris acetate gels (Invitrogen) and transferred onto nitrocellulose membranes. The membranes were treated with a casein-based blocking solution (Western Blocking Reagent, Roche Applied Science) for 1 h at room temperature and then incubated for 1 h with FLAG-tagged Srr2-BR(5 μg/ml) suspended in PBS, 0.05% Tween 20 (PBS-T). The membranes were then washed three times for 15 min in PBS-T, and bound proteins were detected with mouse anti-FLAG antibody (Sigma).

##### Analysis of Srr2-BRs Binding to Fibrinogen by Enzyme-linked Immunosorbent Assay (ELISA)

Purified fibrinogen (0.1 μm) or recombinant fibrinogen truncations were immobilized overnight in 96-well microtiter plates at 4 °C. The wells were blocked with 300 μl of casein blocking solution (Roche Applied Science) for 1 h at room temperature (32, 33). The plates were washed three times with PBS-T and either FLAG-Srr2-BR or FLAG-Srr2-BRΔlatch in PBS-T was added over a range of concentrations for 1 h. The plates were incubated for 1 h at 37 °C, washed with PBS-T to remove unbound protein, and incubated with mouse anti-FLAG antibodies (1:4000) in PBS-T for 1 h at 37 °C. Wells were washed and incubated with HRP-conjugated rabbit anti-mouse IgG (Sigma) diluted 1:5000 in PBS-T for 1 h at 37 °C. For some studies, wells containing immobilized fibrinogen were pretreated with rabbit anti-fibrinogen IgG or recombinant untagged proteins, followed by washing prior to the addition of FLAG-Srr2-BR. Levels of binding were assessed by absorbance at 450 or 495 nm, using 3,3′,5,5′-tetramethylbenzidine or *o*-phenylenediamine dihydrochloride as chromogenic substrates.

##### Lectin Blot Analysis of Srr2 Expression by GBS

Cell wall proteins were released from whole bacteria using mutanolysin, as described previously (4). The proteins were separated by SDS-PAGE in 3–8% Tris acetate gels (Invitrogen) under reducing conditions (0.5 m dithiothreitol) and transferred to nitrocellulose membranes. After incubating for 1 h at room temperature with the casein blocking reagent (Roche Applied Science), the membranes were incubated with biotin-conjugated wheat germ agglutinin (Vector Laboratories) (0.2 μg/ml) followed by incubation with HRP-conjugated streptavidin (0.2 μg/ml) (4).

##### GBS Adherence Assay

Primary hBMEC were purchased from ScienCell (34). Bacterial adherence assays were performed as described (15). In brief, bacteria were grown to mid-log phase and adjusted to the concentration of 10^5^ CFU/ml in PBS. Bacterial suspensions were added to confluent hBMEC monolayers and incubated for 30 min. The wells were washed to remove unbound bacteria and treated with 100 μl of trypsin (2.5 mg/ml) for 10 min at 37 °C to release attached bacteria. The number of bound bacteria was determined by plating serial dilutions of the recovered bacterial suspensions onto THY agar. After 24 h, the number of bacteria were counted, and bacterial adherence was calculated as recovered CFU/initial inoculum CFU × 100%. In the indicated experiments, exogenous fibrinogen (20 μg/ml) was added directly to bacteria and incubated for 30 min with rotation at 37 °C prior to addition to hBMEC monolayers.

##### Binding of GBS to Immobilized Fibrinogen and Recombinant Proteins

Overnight cultures of GBS were harvested by centrifugation and suspended in PBS (final concentration, 10^6^ CFU/ml). Purified fibrinogen (0.1 μm) or recombinant truncated fibrinogen polypeptides were immobilized in 96-well microtiter plates and then incubated with 100 μl of GBS suspension for 30 min at 37 °C. Unbound bacteria were removed from the plates by washing with PBS, and the number of bound bacteria was determined by treating the wells with trypsin and plating serial dilutions of the recovered bacteria onto THY agar plates as described above or staining with crystal violet (0.5% v/v) for 1 min, as described previously (32).

##### Surface Plasmon Resonance (SPR) Spectroscopy

SPR spectroscopy was performed using a BIAcore T100 system (GE Healthcare). Purified human fibrinogen (10 μg/ml in sodium citrate buffer, pH 5.5) was covalently immobilized on CM5 sensor chips using amine coupling as described previously (35, 36). Increasing 2-fold concentrations (range, 1.25–160 μm) of Srr1-BR and Srr2-BR were flowed over fibrinogen or block reagent (ethanolamine) at a rate of 10 μl/min. The sensorgram data were subtracted from the corresponding data from the reference flow cell and analyzed using the BIAevaluation software version 3.0. A plot of the level of binding (response units) at equilibrium against a concentration of analyte was used to determine the *K_D_*.

##### Isothermal Titration Calorimetry (ITC)

ITC was performed with a MicroCal ITC_200_ microcalorimeter at 25 °C as described previously for ClfA (a fibrinogen-binding protein of *S. aureus*) (35). All recombinant proteins were dialyzed against HBS buffer (10 mm HEPES, 150 mm NaCl, pH 7.4). The reaction cell contained 50 μm fibrinogen Aα RU678 or RU789 (expressed as MBP fusion proteins), and the syringe contained 0.5 mm recombinant Srr1-BR or Srr2-BR in HBS buffer. These concentrations were based on the above-published studies with ClfA. The data were analyzed using MicroCal Origin software (version 5.0), with results fitted to a single binding mode (35, 36).

##### Crystallization of Srr Binding Regions

Purified recombinant Srr1-BR (7.6 mg/ml in 0.5 m NaCl, 0.01 m Tris-HCl, pH 8.3, 5 mm β-mercaptoethanol) was crystallized using the sitting drop vapor diffusion method with 1 μl of protein incubated with 0.2 mm peptide (NPGSPRPGSTGTWNPGSSERGSAGHWTSESSVSGSTGQWHSESGSFRPDSPG) and 1 μl of reservoir solution (0.2 m CaCl_2_, 0.1 m Tris-HCl, pH 6.0, 20% (w/v) PEG 6000) at room temperature. Srr2-BR was crystallized using the sitting drop vapor diffusion method with 1 μl of protein (7.5 mg/ml, 0.25 m NaCl, 0.01 m Tris-HCl, pH 8.3, 5 mm β-mercaptoethanol) and 1 μl of reservoir solution (5 m NaCl) at room temperature.

Crystals of *S. agalactiae* Srr1-BR were cryo-cooled from the reservoir solution without additional cryoprotectant. Data were collected at 100 K using beamline 21-ID-D of the Life Sciences Collaborative Access Team (LS-CAT) at the Advanced Photon Source (Argonne, IL) using a wavelength of 0.97928 Å and a MarMosaic 300 CCD detector. Crystals of *S. agalactiae* Srr2-BR were removed from the crystallization droplet and cryo-protected in 4 m NaHCOO prior to cryo-cooling. Data were collected at 100 K on beamline 21-ID-G of the LS-CAT using a wavelength of 0.97956 Å and a MarMosiac 300 CCD detector. All data were processed using the HKL3000 suite (37). The structure of Srr1-BR was determined by molecular replacement using PHASER (38) and the structure of *S. epidermidis* adhesin SdrG (Protein Data Bank code 1R17) as a search model (39). The structure of Srr2-BR was determined by molecular replacement with the program PHASER (38, 40) using the refined coordinates of Srr1 as the search model. Both models were improved using iterative rounds of model building in COOT and refinement in REFMAC (41, 42). Details of data collection, structure determination, refinement, and model quality are provided in Table 3.

**TABLE 3:** **Crystallographic data collection and refinement statistics**

	*S. agalactiae* Srr1-BR	*S. agalactiae* Srr2-BR
PDB entry	4MBO	4MBR

**Data collection**		
Resolution	30 to 1.65 Å (1.68 to 1.65 Å)*^a^*	30 to 3.65Å (3.71 to 3.65 Å)
Beamline	21-ID-D	21-ID-G
Wavelength	0.97928 Å	0.97956 Å
Space group	P2_1_2_1_2	P4_1_2_1_2
Unit cell parameters	*a* = 73.36Å	*a* = *b* = 97.60 Å
	*b* = 83.24 Å	
	*c* = 57.06 Å	*c* = 173.27 Å
	α = β = γ = 90°	α = β = γ = 90°
No. of reflections	255,636	93,050
Unique reflections	42,747	9,911
Completeness	99.5% (100.0%)	99.9% (100.0%)
*I*/σ	26.4 (3.8)	41.0 (3.3)
*R*_sym_*^b^*	0.058 (0.500)	0.050 (0.690)

**Refinement**		
*R*_work_*^c^*	0.154	0.213
*R*_free_*^c^*	0.187	0.241
No. of *R*_free_ reflections	2141	471

*^a^* Values in parentheses are for the highest resolution shell.

*^b^ R*_sym_ = Σ(*I_i_* − 〈*I*〉)/Σ (〈*I*〉), where *i* is the *i*th measurement and 〈*I*〉 is the weighted mean of *I*.

*^c^ R*_work_ = Σ ‖*F*_obs_| − |*F*_calc_‖/*S*|*F*_obs_|.

*^d^ R*_free_ is calculated using the same equation as *R*_work_ using a subset of reflections omitted from refinement and reserved in the test set of the data.

##### Data Analysis

Binding data are expressed as means ± S.D. and were compared for statistical significance by the unpaired *t* test.

## RESULTS

### 

#### 

##### Srr2 Mediates GBS Binding to Fibrinogen

**FIGURE 1.**
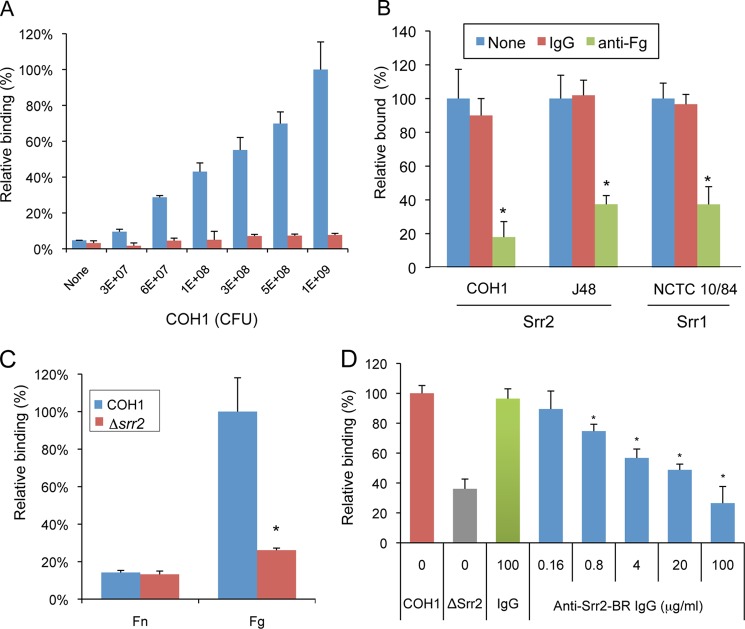
**GBS binding to fibrinogen is mediated by glycoprotein Srr2.** *A,* suspensions of GBS strain COH1 were incubated in microtiter wells with immobilized fibrinogen (*blue*) or a casein-based blocking reagent (*red*). Binding of bacteria was assessed by crystal violet staining and expressed as the means ± S.D. absorbance. *B,* immobilized fibrinogen was pre-incubated with anti-rabbit fibrinogen (*Fg*) IgG (100 μg/ml) or rabbit IgG (100 μg/ml) prior to testing for binding by GBS strains. Unbound IgG was removed by washing, and GBS binding was assessed. Values represent percent of GBS binding, as compared with untreated fibrinogen. NCTC 10/84, which expresses Srr1, served as a control. *C,* binding of GBS strain COH1 or its *srr2* variant (Δ*srr2*) to immobilized to fibronectin (*Fn*) or fibrinogen (*Fg*). *D,* inhibition of GBS COH1 binding to fibrinogen by anti-Srr2 IgG. Strain COH1 was co-incubated with rabbit anti-Srr2 IgG or normal rabbit IgG, and relative binding to immobilized fibrinogen was assessed. Values are mean ± S.D. of relative binding, normalized for WT levels of binding to fibrinogen. *, *p* < 0.01.

We have previously shown that the Srr1 glycoprotein of GBS can bind fibrinogen *in vitro* and that this interaction mediates bacterial binding to the host *in vivo* (4). To assess whether Srr2 has a similar role, we first measured the adherence of two GBS strains expressing Srr2 to immobilized fibrinogen. Strain NCTC 10/84, which expresses Srr1, served as a control for fibrinogen binding. As shown in Fig. 1*A*, strains COH1 adhered to immobilized human fibrinogen at levels that were significantly higher than those seen with a negative control (casein). Similar results were seen with strain J48 (data not shown). Binding of the strains was significantly inhibited by pretreatment of immobilized fibrinogen with anti-fibrinogen IgG, indicating that the interaction between GBS and fibrinogen was specific (Fig. 1*B*).

To more directly assess the impact of Srr2 on bacterial binding, we compared the binding of COH1 and an Srr2-deficient strain (COH1Δ*srr2*) to immobilized fibrinogen. As shown in Fig. 1*C*, loss of *srr2* expression significantly reduced GBS binding to fibrinogen but had no effect on bacterial binding to immobilized fibronectin. Expression of the *srr2* gene in *trans* restored binding to wild type (COH1) levels, demonstrating that the loss of binding observed with *srr2* disruption was not due to polar or pleiotropic effects (Fig. 9*B*). In addition, binding by COH1 to fibrinogen was inhibited by rabbit anti-Srr2 IgG but not by normal (preimmune) rabbit IgG (Fig. 1*D*). The level of inhibition was concentration-dependent, with 100 μg/ml of anti-Srr2 IgG being sufficient to reduce WT GBS binding to levels comparable with those seen with GBSΔ*srr2*. These results indicate that the binding of GBS COH1 to immobilized fibrinogen is predominantly mediated by Srr2.

**FIGURE 2.**
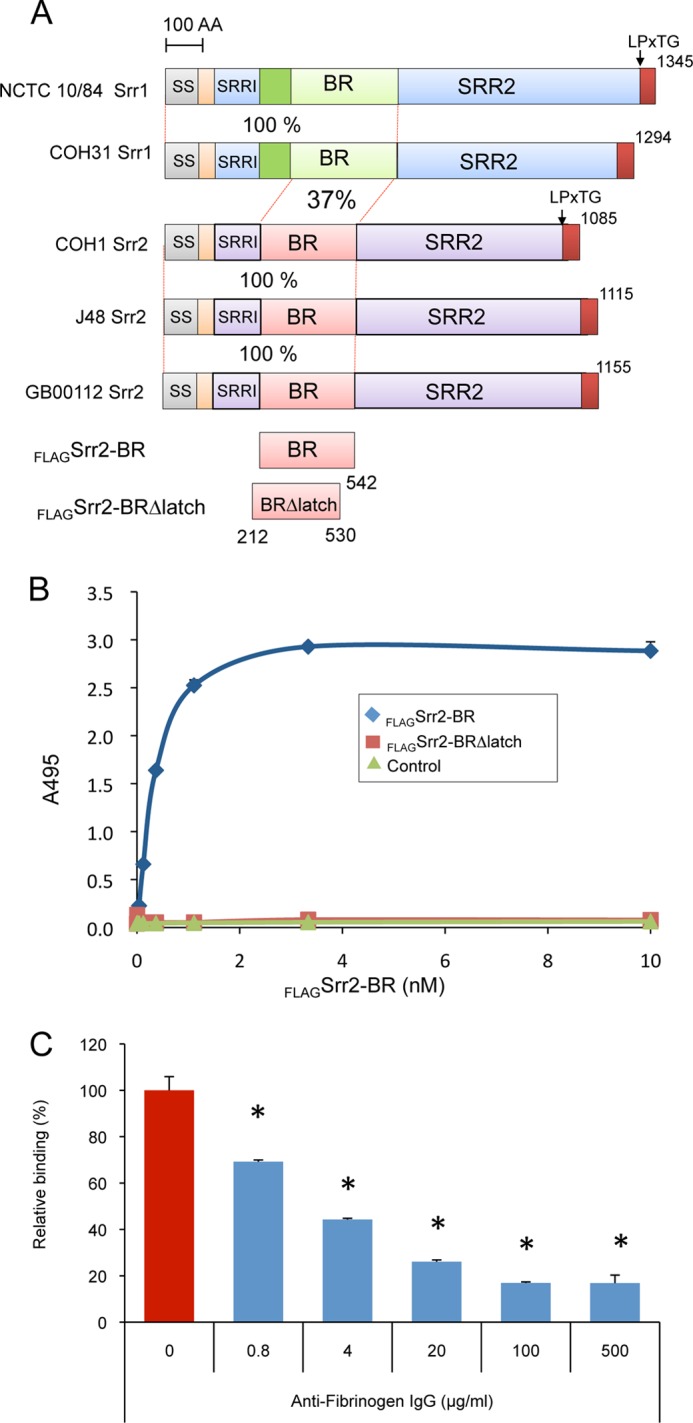
**Interaction of the BR of Srr2 with fibrinogen.** *A,* schematic diagram of the serine-rich repeat proteins Srr1 and Srr2. Level of identity (%) between regions is indicated. *SS,* signal sequence; *Srr1-BR* and *Srr2-BR*, binding domains; *SRR1* and *SRR2,* serine-rich regions; *LPxTG*, cell wall anchoring motif; *AA,* amino acids. *B,* binding of FLAG-Srr2-BR and FLAG-Srr2-BRΔlatch proteins to immobilized fibrinogen. Indicated concentrations of FLAG-Srr2-BR and FLAG-Srr2-BRΔlatch were added to wells coated with fibrinogen or casein blocking reagent. *C,* inhibition of FLAG-Srr2-BR binding to immobilized fibrinogen by anti-fibrinogen IgG. Values represent percent of FLAG-Srr2-BR binding to the wells treated with fibrinogen. *Bars* indicate the means ± S.D. *, *p* < 0.01.

To confirm that the putative binding region of Srr2 interacts with fibrinogen, we assessed the interaction of the purified FLAG-tagged binding region (FLAG-Srr2-BR) with immobilized human fibrinogen (Fig. 2). In control studies, no significant binding by FLAG-Srr2-BR to immobilized casein was detected. In contrast, FLAG-Srr2-BR showed significant levels of binding to fibrinogen, which increased in direct proportion to the amount of protein applied. At concentrations above 3.3 μm of FLAG-Srr2-BR, binding reached a plateau, consistent with saturation. Binding of Srr2-BR was significantly inhibited by anti-fibrinogen IgG, indicating that this interaction was specific (Fig. 2*C*).

##### Structures of S. agalactiae Srr1 and Srr2 Binding Regions

**FIGURE 3.**
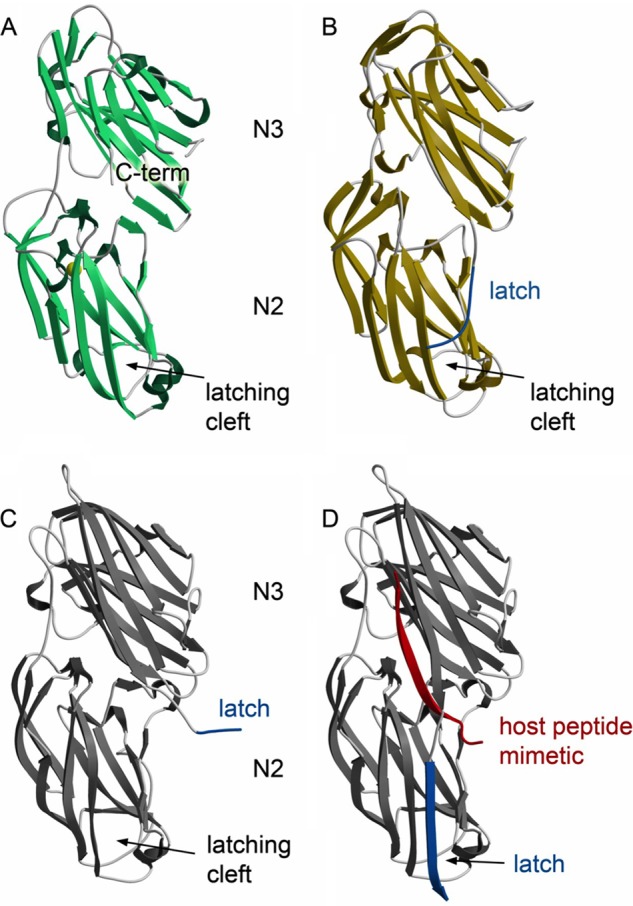
**Structures of Srr1-BR and Srr2-BR.** *A,* structure of Srr1-BR with secondary structural elements colored *green* and turns colored in *gray*. The latch region is disordered in the structure. *B,* structure of Srr2-BR with secondary structural elements colored *yellow* and turns colored *gray*. The latch region is highlighted in *cerulean blue. C,* structure of ClfB without peptide ligand mimetic shows a latching region that is open and an unoccupied latching cleft ((Protein Data Bank entry 4F24 (19)). The ordered region of the latch is highlighted in *cerulean blue. D,* structure of ClfB with peptide ligand mimetic identifies peptide binding to the cleft between the N2 and N3 domains and shows the latch in the locked position. (Protein Data Bank entry 4F27 (19)). The peptide is shown in *red,* and the latch is shown in *cerulean blue*.

To assess whether Srr1 and Srr2 could support a DLL mechanism of ligand binding, we determined the crystal structures of *S. agalactiae* Srr1-BR and Srr2-BR at resolutions of 1.65 and 3.65 Å, respectively (Fig. 3). As was previously predicted from sequence and functional analyses (4), Srr1-BR and Srr2-BR each adopt an overall fold that resembles the binding regions of “microbial surface components recognizing adhesive matrix molecules” (MSCRAMMs), including the well characterized ClfA (43, 44), ClfB (20), SdrG (21), and the likely MSCRAMM UafA (45). However, Srr1-BR and Srr2-BR are more similar to each other than they are to these structurally characterized MSCRAMMs, with a root mean square deviation of 1.6 Å between Srr1-BR and Srr2-BR and root mean square deviations between 2.1 and 2.5 Å when either Srr1 or Srr2 is placed in a pairwise structural alignment with ClfA, ClfB, SdrG, or UafA (20, 21, 43–45).

Like other MSCRAMMs, Srr1-BR and Srr2-BR contain two domains, termed N2 and N3, with each adopting the DE variation of the Ig fold (44). Between the N2 and N3 domains is a cleft, demonstrated to be the ligand-binding site in other MSCRAMMs. The size and shape of the interdomain cleft is consistent with this being the binding site. An unusual feature of the Srr2-BR structure, not observed in Srr1-BR or in any previous structures of MSCRAMMs, is the conformation of the C terminus of the N3 domain. To date, the structures of MSCRAMMs crystallized in the absence of a ligand have the C-terminal extension of the N3 domain either completely or partially disordered (21, 44, 45). This C-terminal extension, known as the latch, has been shown to close over the peptide mimetic of a host ligand in co-crystal structures. Once closed, the latch forms a β-strand that completes a fully hydrogen-bonded β-sheet within the N2 domain and locks the host ligand in place (21, 43) (Fig. 3, *C* and *D*). In contrast to other structurally characterized DLL proteins, the latch region in *S. agalactiae* Srr2-BR adopted a distinct conformation in the absence of ligand. Although the assignment of amino acids is somewhat tenuous at this resolution, electron density is consistent with the latch being nearly closed or “ajar.”

##### Srr2-BR Binding to Fibrinogen Occurs through a Variant DLL Mechanism

**FIGURE 4.**
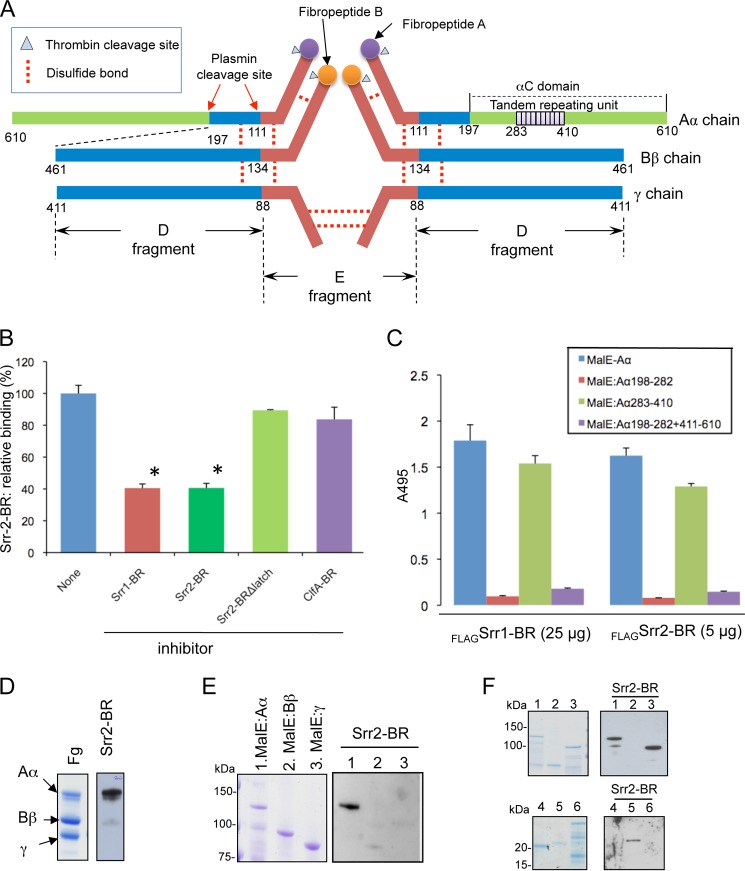
**Identification of the Srr2 binding domain on fibrinogen.** *A,* schematic drawing of human fibrinogen. The 10 tandem repeating units of the Aα chain are shown in *purple. B,* inhibition of FLAG-Srr2-BR (0.05 μm) binding to immobilized fibrinogen by purified untagged proteins (10 μm). *C,* binding of FLAG-Srr1-BR (*_FLAG_Srr1-BR*) (25 μg/ml) or FLAG-Srr2-BR (*_FLAG_Srr1-BR* (5 μg/ml) to MBP fused with full-length recombinant Aα (MBP-Aα) or subdomains of the Aα chain. *Subscripts* indicate amino acids contained within each fragment. *Bars* represent the mean binding levels (± S.D.). *D,* Srr2-BR binding to the fibrinogen (*Fg*) Aα chain. Purified human fibrinogen was separated by SDS-PAGE and stained with Coomassie Blue (*left panel*). Far Western blotting of fibrinogen with Srr2-BR is shown in the *right panel. E,* recombinant MBP-Aα, Bβ, and γ chains probed with FLAG-Srr2-BR (5 μg/ml). *F,* recombinant MBP-Aα and its truncated variants probed with FLAG-Srr2-BR (5 μg/ml; *right*). *Lane 1*, MBP:Aα(1–610); *lane 2*, MBP:Aα(1–197); *lane 3*, MBP:Aα(198–610); *lane 4*, MBP:Aα(198–282); *lane 5*, MBP:Aα(283–410); and *lane 6*, MBP:Aα(198–282 + 411–610).

To reveal whether the latch domain of Srr2-BR was important for binding, we generated a variant of the protein (Srr2-BRΔlatch), in which the terminal 13 residues of this domain were deleted. We have previously shown that this type of deletion in Srr1-BR abrogates binding, without altering the overall conformation of the protein (4). As shown in Fig. 2*B*, removing the C-terminal amino acids of the Srr2 binding region abolished fibrinogen binding. In addition, untagged Srr2-BR inhibited the binding of FLAG-Srr2-BR to immobilized fibrinogen, whereas untagged Srr2-BRΔlatch failed to block this interaction (Fig. 4*B*). These data indicate that the fibrinogen binding domain of Srr2 is indeed located in the predicted binding region and that Srr2-BR binds fibrinogen by a DLL mechanism.

**FIGURE 5.**
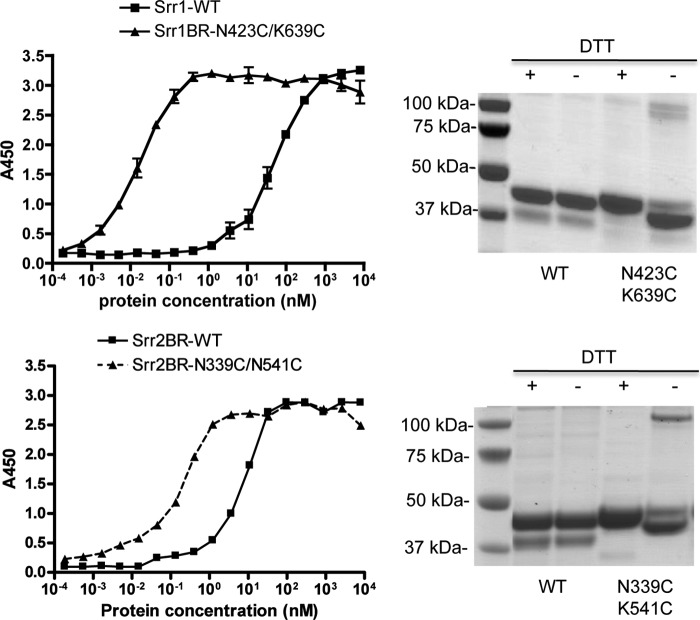
**Effect of cross-linking the latch and latching cleft domains on fibrinogen binding.** *Left panels,* cysteine substitutions were made in the indicated residues of Srr1-BR and Srr2-BR, and the resultant recombinant proteins were compared with their respective WT proteins for binding to immobilized fibrinogen, as measured by ELISA. Values shown are the means ± S.D. *Right panels,* SDS-PAGE of WT *versus* cysteine-cross-linked Srr1-BR and Srr-2 BR under reducing (+) or nonreducing (−) conditions (Coomassie Blue staining).

Based on the above findings, we hypothesized that the affinity of Srr-BRs to fibrinogen is affected by the location of the latch prior to ligand binding, with a more closed conformation of the latch associated with enhanced affinity. To address this possibility, we constructed variants of the Srr1 and Srr2 BRs, in which residues in both the latch and latching cleft regions were replaced with a cysteine, such that a disulfide bond would be formed, thus fixing the spatial position of the latch. We used the crystal structures to guide the location of the cysteine insertions, to generate BRs with the latch adopting a closed conformation, even in the absence of ligand. When assessed by SDS-PAGE under nonreducing conditions, the mutated Srr1 and Srr2 BRs migrated faster than their respective WT proteins, presumably due to the more compact folding of the cross-linked proteins (Fig. 5). Under reducing conditions (1 mm DTT), the mutant proteins had mobilities identical to their WT counterparts As compared with the WT Srr1-BR and the Srr2 BR, both cross-linked proteins showed enhanced binding to fibrinogen (Fig. 5). These data indicate that the affinities of Srr1 and Srr2 for fibrinogen are enhanced by a pre-closed latch, consistent with the location of the latch influencing binding affinity.

##### Identification of the Fibrinogen Region Bound by Srr2-BR

Fibrinogen is a 340-kDa hexameric glycoprotein composed of three pairs of chains (α, β, and γ) linked by disulfide bonds (Fig. 4*A*). To identify the binding site on fibrinogen for Srr2, we first examined whether binding of FLAG-Srr2-BR to immobilized fibrinogen could be inhibited by untagged Srr1-BR (which binds the tandem repeat region of the fibrinogen Aα chain) or untagged ClfA-BR (which binds the N terminus of the fibrinogen Bβ chain) (4, 46). As shown in Fig. 4*B*, binding of FLAG-Srr2-BR was significantly reduced by pretreatment of immobilized fibrinogen with either untagged Srr1-BR or Srr2-BR. In contrast, no inhibition was seen with ClfA-BR. To further characterize the binding site for Srr2 on fibrinogen, we analyzed by far Western blotting the interaction of FLAG-Srr2-BR with human fibrinogen and recombinant fibrinogen Aα, Bβ, and γ chains. As was found for Srr1 (4), Srr2 binding was only detected to the Aα chain (Fig. 4, *C* and *D*). To better define the binding site on this chain for the SRR proteins, we then measured their binding to recombinant Aα chain fragments. When assessed by ELISA, we found no significant binding of either FLAG-Srr1-BR or FLAG-Srr2-BR to MBP:Aα(198–282) or MBP:Aα(198–282 + 411–610). In contrast, both SRR proteins exhibited levels of binding to MBP:Aα(283–410) that were comparable with those seen with the recombinant full-length Aα chain (MBP:Aα(1–610); Fig. 4*E*). Far Western blotting analysis confirmed that the Srr1-BR- and Srr2-BR-binding sites are indeed contained within the tandem repeat region (amino acids 283–410) of the Aα chain of fibrinogen (Fig. 4*F*).

**FIGURE 6.**
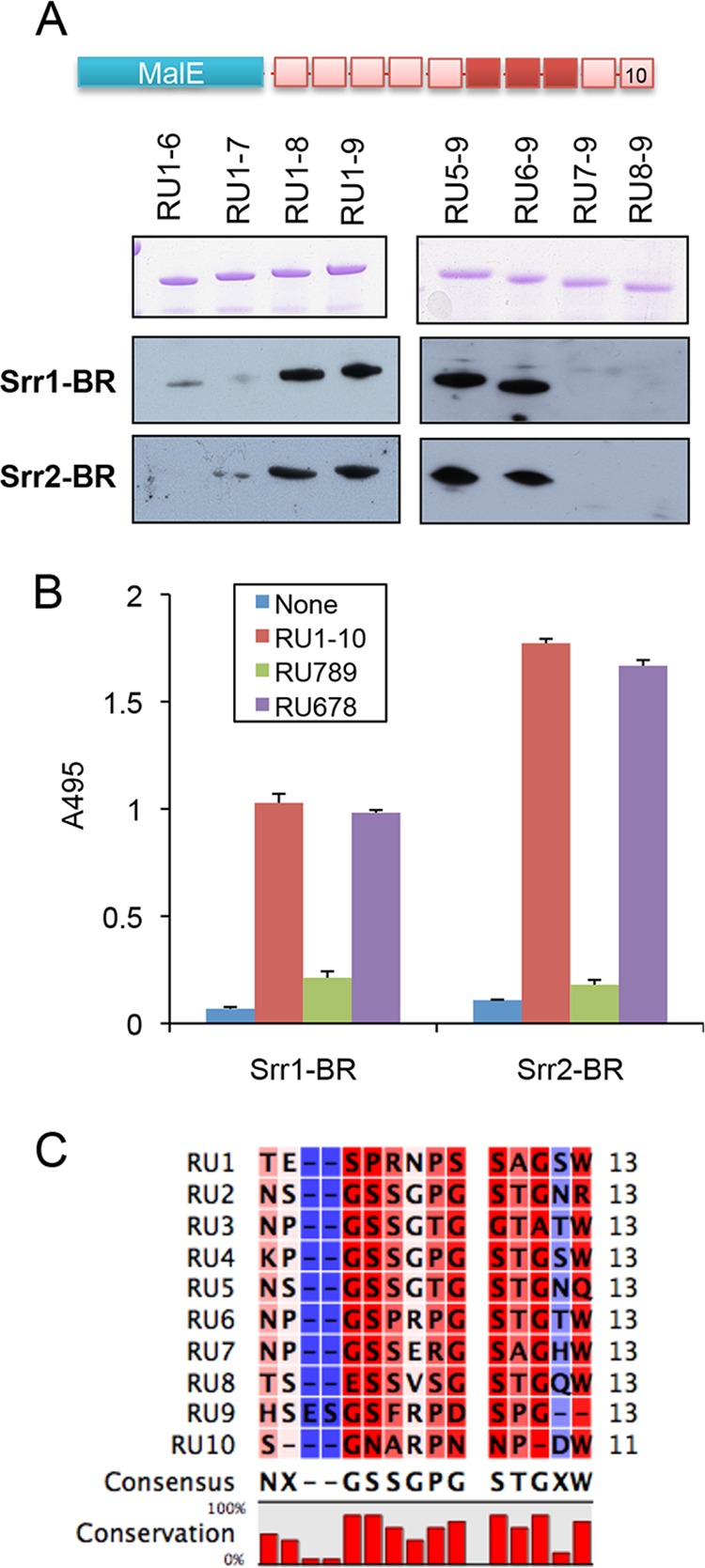
**Srr1-BR and Srr2-BR bind the repeat domain of the fibrinogen Aα chain.** *A,* recombinant MBP-RU fusion proteins were separated by SDS-PAGE and stained with Coomassie Blue (*top*) or transferred to nitrocellulose and probed with FLAG-Srr1-BR (25 μg/ml, *middle*) or FLAG-Srr2-BR (5 μg/ml, *bottom*). *B,* FLAG-Srr1-BR or FLAG-Srr2-BR (5 μg/ml) was incubated with immobilized recombinant MBP-RU1–10, MBP-RU789, or MBP-RU6789. Binding was detected by ELISA using anti-FLAG antibody. *Bars* indicate the means (± S.D.).

We then sought to characterize further which subdomain within the tandem repeat region is the receptor for the SRR proteins. This region of the fibrinogen Aα chain is composed of 10 repeating units, each containing 13 amino acids (Fig. 6). We therefore expressed various portions of this region as maltose-binding protein fusions and assessed SRR protein binding to these peptides by far Western blotting (Fig. 6*A*). Peptides composed of repeat units 1–8 (RU1–8) and 1–9 (RU1–9) were bound by both Srr1-BR and Srr2-BR. In addition, tandem repeat units 5–9 (RU5–9) and 6–9 (RU6–9) were bound by both binding regions, indicating that the binding site of the Srr proteins is located within tandem repeats 6–8 of the fibrinogen Aα chain. To directly confirm these findings, we assessed Srr1-BR or Srr2-BR binding to peptides composed of tandem repeat units 1–10 (RU1–10), 6–8 (RU678), and 7–9 (RU789), as measured by ELISA (Fig. 6*B*). Binding levels of Srr1-BR and Srr2-BR to RU678 were comparable with those seen with the full-length tandem repeat region (RU1–10). In contrast, no binding was detected with immobilized RU789, suggesting that the RU678 comprises the minimum target for Srr1-BR and Srr2-BR binding to the fibrinogen Aα chain.

**FIGURE 7.**
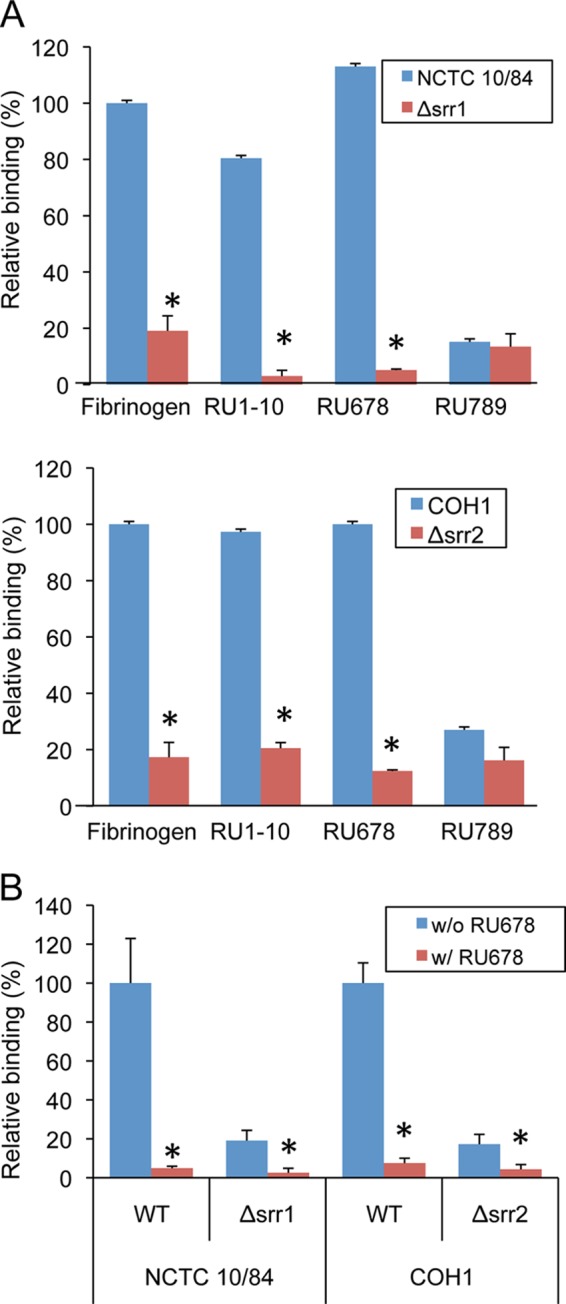
**GBS binds RU678 of the fibrinogen Aα chain.** *A,* GBS strains NCTC 10/84 and COH1 were compared with their Δ*srr1* or Δ*srr2* isogenic variants for binding to fibrinogen, MBP-RU1–10, MBP-RU678, and MBP-RU789. Values are levels of binding relative to the WT strain, expressed as mean ± S.D. *, *p* < 0.01. *B,* inhibition of GBS COH1 or NCTC 10/84 binding to fibrinogen by purified MBP-RU678. 10^6^ CFU of WT GBS were co-incubated with MBP-RU678 in 96-well plates coated with fibrinogen. Values represent percent of WT GBS binding. *, *p* < 0.01.

To examine whether the binding of GBS NCTC 10/84 and COH1 to fibrinogen was mediated by the interaction of Srr1-BR and Srr2-BR with RU678, the above WT strains and their respective Δ*srr1* or Δ*srr2* isogenic mutants were incubated with immobilized fibrinogen, RU1–10 (amino acids 283–410), RU678, or RU789 (Fig. 7). The WT GBS strains exhibited levels of binding to RU678 that were comparable with those seen with fibrinogen, but both strains had only minimal levels of binding to immobilized RU789. In contrast, the GBSΔ*srr1* and Δ*srr2* mutant strains exhibited low levels of binding to all immobilized proteins. To further investigate whether GBS binding to fibrinogen was mediated by the interaction of Srr1-BR or Srr2-BR with RU678, we tested the ability of the RU678 peptide to inhibit GBS binding to immobilized fibrinogen. Preincubation of GBS strains with 10 μm RU678 resulted in a significant reduction in binding (Fig. 7*B*), further indicating that bacterial binding to fibrinogen is mediated by the interaction of the Srr proteins with repeating units 6–8 of the fibrinogen Aα chain.

##### Quantitative Assessment of SRR Protein Binding to Fibrinogen by SPR and ITC

**FIGURE 8.**
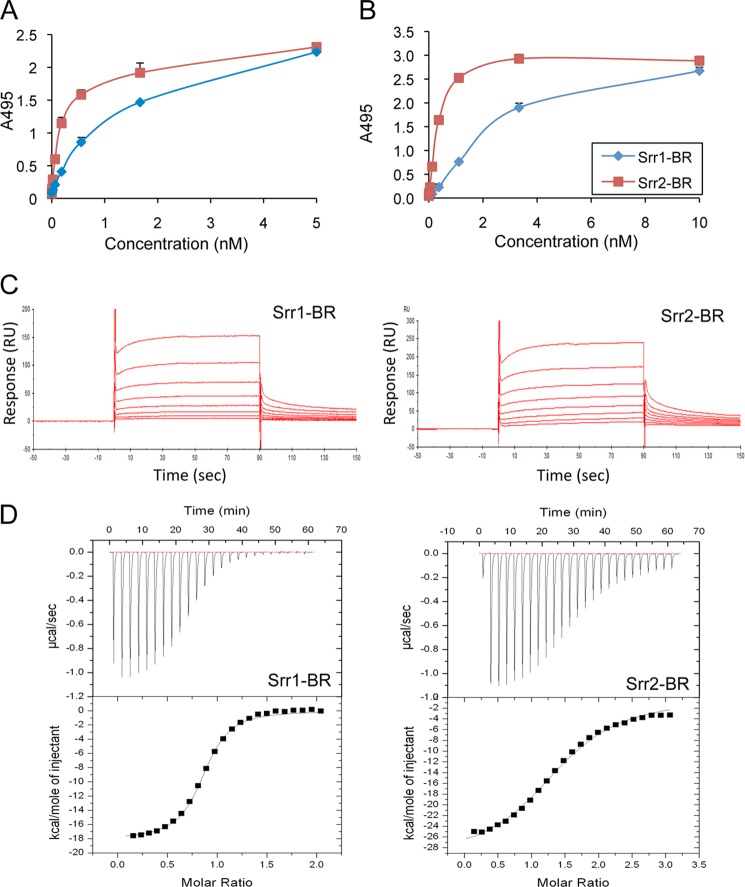
**Interaction of the Srr binding regions with fibrinogen and RU678.** Binding is shown of purified Srr1-BR and Srr2-BR proteins (FLAG-Srr1-BR and FLAG-Srr2-BR) to immobilized fibrinogen (*A*) or MBP-RU678 (*B*). Bound proteins were detected with anti-FLAG antibody. *Bars* indicate the means (± S.D.). No binding was seen to casein-coated wells (data not shown). *C,* surface plasmon resonance analysis of SRR binding to fibrinogen. Sensorgrams of binding to fibrinogen were obtained by passing 1.25–160 μm of Srr1-BR (*A*) or Srr2-BR (*B*) over fibrinogen immobilized on the surface of a CM5 sensor chip. Injections began at 0 s and ended at 90 s. The results shown are representative of two independent experiments. *D,* ITC analysis of Srr-BR binding to RU678. Srr1-BR (*A*) or Srr2-BR (*B*) were injected into an ITC chamber containing MBP-RU678. The *upper panels* show enthalpic heat released per s at 25 °C during titration, and the *lower panels* show integrated binding isotherms of the titration fitted to a one-site model.

We assessed the relative binding of Srr1-BR and Srr2-BR to immobilized fibrinogen (0.1 μm) or MBP-RU678 (0.1 μm), by ELISA as described previously (4). As shown in Fig. 8, *A* and *B*, both proteins bound to immobilized fibrinogen and MBP-RU678 in a concentration-dependent manner. Next, the binding of Srr1-BR and Srr2-BR to fibrinogen was analyzed via SPR (Fig. 8*C*). Increasing concentrations of Srr1-BR or Srr2-BR (1.25–160 μm) were flowed over fibrinogen immobilized on a CM5 chip, and the dissociation constant (*K_D_*) of binding was determined from analysis of the equilibrium binding data. Srr1-BR and Srr2-BR showed specific and concentration-dependent binding to human fibrinogen. The *K_D_* values of Srr1-BR and Srr2-BR were determined to be 2.1 × 10^−5^ and 3.7 × 10^−6^ m, respectively. These values are within the range reported for fibrinogen-binding proteins of Gram-positive bacteria (43, 47). However, the above *K_D_* value for Srr1-BR was considerably larger than the 7.51 × 10^−8^ we had previously calculated, based on ELISA data (4). A similar variation in *K_D_* values has been reported for ClfA binding to fibrinogen (43), although the reason for this variability is unclear.

We next sought to confirm these results by ITC. Because of the limited solubility of fibrinogen, we were unable to assess its binding to the Srr binding regions by this method. For that reason, we assessed the binding of Srr1-BR and Srr2-BR to RU678 (Fig. 8*D*). In control studies, no significant interaction of Srr1-BR or Srr2-BR with RU789 was detected (data not shown). However, Srr1-BR and Srr2-BR bound RU678 with dissociation constants (*K_D_*) of 6.9 × 10^−5^ and 1.2 × 10^−5^ m, respectively. Both binding reactions were exothermic, and the stoichiometry (*n*) of the binding reaction with both proteins was close to 1.

##### GBS Strains Expressing Srr2 Have Higher Levels of Fibrinogen and Endothelial Cell Binding

**FIGURE 9.**
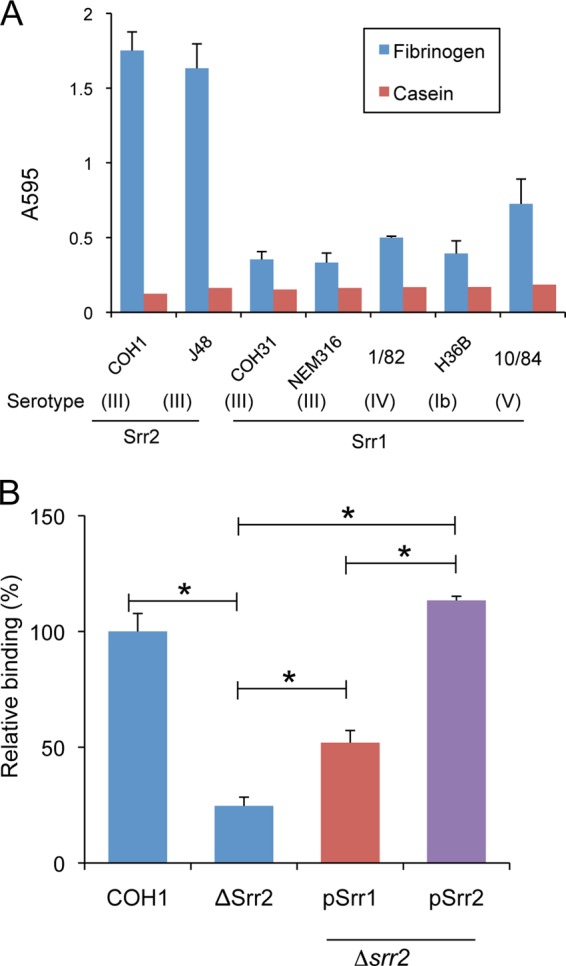
**GBS binding to immobilized fibrinogen.** *A,* GBS strains were incubated with wells pre-treated with fibrinogen (0.1 μm) or a casein blocking reagent. *B,* fibrinogen binding by strain COH1, COH1Δ*srr2* (“Δ*srr2*”), and the mutant complemented with a plasmid encoding either *srr1* (*pSrr1*) or *srr2* (*pSrr2*). *, *p* < 0.01.

Because Srr2-BR exhibited higher binding affinity to fibrinogen than Srr1-BR, we next compared fibrinogen binding by Srr1-expressing strains with strains expressing Srr2. As shown in Fig. 9, the Srr2 strains (COH1 and J48) had significantly higher levels of fibrinogen binding, as compared with five strains expressing Srr1. We then examined the impact of expressing either Srr1 or Srr2 in strain COH1Δ*srr2* (Fig. 9*B*). Of note, expression levels of the SRR glycoproteins on the cell surface of the complemented strains were comparable, as measured by binding to wheat germ agglutinin, although somewhat lower than those seen with the WT strain (data not shown). Complementation with the *srr1* gene in *trans* significantly increased fibrinogen binding by COH1 Δ*srr2*, but not to levels observed with the WT strain. However, complementation of the same mutant with *srr2* gene in *trans* restored binding of COH1 Δ*srr2* to WT levels. These results indicate that the higher affinity of Srr2 for fibrinogen, as compared with Srr1, can result in higher levels of bacterial binding to the protein.

**FIGURE 10.**
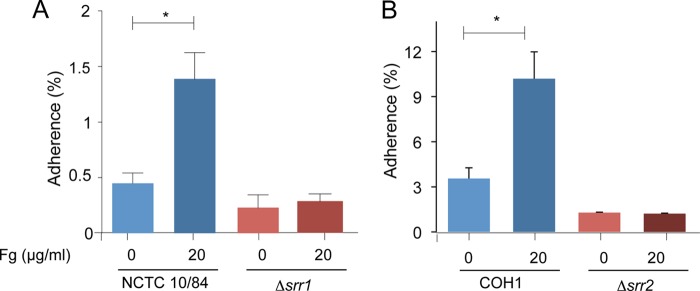
**GBS adherence to hBMEC is mediated by the interaction of the Srr protein and fibrinogen.** GBS strains NCTC10/84 (*A*) or COH1 (*B*) or their Δ*srr* mutants were incubated with hBMEC, with or without fibrinogen pretreatment (20 μg/ml). Values represent percent (mean ± S.D.) of inoculum bound to the monolayers. *, *p* < 0.01.

The attachment of GBS to hBMEC is thought to be important for the invasion of the organism into the central nervous system (48, 49). Our previous study indicates that Srr1-fibrinogen binding is important for the attachment of GBS to hBMEC. To determine whether Srr2 has a similar role, we assessed the impact of Srr2 on GBS attachment to hBMEC pretreated with purified fibrinogen. WT GBS and isogenic Δ*srr1* and Δ*srr2* variants were incubated with hBMEC. After 30 min, WT GBS efficiently adhered to these cells, whereas the Δ*srr1* and Δ*srr2* mutants were significantly reduced in binding (*p* < 0.01) (Fig. 10). Preincubation of bacteria with purified human fibrinogen (20 μg/ml) enhanced the binding of the WT strains to hBMEC but had no effect on the binding of the Δ*srr1* and Δ*srr2* mutant strains. Of note, strain COH1, which expresses Srr2, had higher levels of binding to hBMEC, as compared with the Srr1-expressing strain (NCTC 10/84), which was further increased by the addition of fibrinogen.

## DISCUSSION

*S. agalactiae* is a leading cause of neonatal bacteremia and meningitis. Infection is initiated by colonization of the lower genital tract of pregnant women, followed by bacterial invasion and neonatal involvement. Srr1 has been shown to enhance the attachment of bacteria to vaginal and cervical epithelial cells *in vitro* and to augment genital colonization in mice (17). In addition, expression of the protein is associated with increased pathogenicity in animal models of infection. This enhanced virulence appears to be due at least in part to the binding of fibrinogen via Srr1, resulting in increased microvascular invasion and CNS penetration.

In contrast, Srr2 is associated with hypervirulence, but the ligand for this adhesin was previously unknown. Although the binding regions of Srr1 and Srr2 have limited amino acid sequence homology, our findings demonstrate that Srr2 also binds human fibrinogen. In addition, the crystal structures of the binding regions of Srr1 and Srr2 indicate that these both resemble those of several other fibrinogen-binding proteins, including ClfA and ClfB of *S. aureus*, and SdrG of *S. epidermidis* (19, 21, 43, 50). These and a number of other adhesins of Gram-positive bacteria bind fibrinogen through a DLL-like mechanism (35, 47, 51, 52). Co-crystal structures of MSCRAMMs with peptide mimetics of host ligands have revealed that an extended conformation of the polypeptide binds within a cleft between the N2 and N3 domains and forms a β-strand that hydrogen bonds to (and completes) a β-sheet of the N3 domain. This docking of host peptide to the trench in turn induces the C-terminal extension of the MSCRAMM to fold over the host ligand and lock it down. Variations of the DLL mechanism include whether the MSCRAMM has an inactivated state that requires a conformational change prior to binding the host ligand, whether the host ligand binds parallel or antiparallel to the strands of the MSCRAMM, and whether the latch can be closed before (latch and dock) or after (dock, lock, and latch) binding of the peptide mimetic of the host ligand (20, 21, 35, 39, 43, 51).

Srr1 and Srr2 appear to bind fibrinogen by a DLL process, because these share a fold with DLL proteins and because deletion of the predicted latch domains of the SRR proteins significantly reduced fibrinogen binding. As noted above, a shared feature of DLL adhesins is the binding of fibrinogen through a trench formed between two IgG-like folds within the binding domain. Although sequence alignment of the binding regions of Srr1 and Srr2 revealed little homology between their putative binding trenches or with the binding subdomain of ClfA (data not shown), the structures of Srr1 and Srr2 suggest that the binding trenches are more similar to each other than to other DLL proteins, consistent with the trench sequence conferring ligand selectivity. Notably, a bulky amino acid of the N3 domain (Tyr-623 of Srr1 and His-528 of Srr2) significantly constricts the center of each trench. In other structurally characterized DLL proteins, this location harbors a conserved asparagine.

One interesting finding is that, although the Srr proteins interact with the same region of fibrinogen, the binding affinity of Srr2 was higher, both when measured by SPR (using whole fibrinogen as the ligand) and by ITC (using recombinant tandem repeats 6–8). Examination of the structures of the *S. agalactiae* Srr1 and Srr2 binding regions suggests how Srr2 might have greater affinity for the host ligand. In Srr2, the C-terminal latch of N3 appears to be pre-ordered in a conformation that might best be called “open, but ajar” that is poised to close over host ligand quickly. Experimentally reported time scales for protein conformational changes can vary widely, and the time scales of the conformational changes in Srr1 and Srr2 have not been measured. Although the cysteine cross-linking studies suggest that both Srr1 and Srr2 C termini are able to close even in the absence of ligand, the pre-ordered conformation of the C terminus in Srr2 would almost certainly require less time to do so. This would theoretically increase the *k*_on_ and reduce the *k*_off_, and it could be numerically reflected as improved ligand affinity. This is consistent with previous studies of the MSCRAMM ClfA (43), where the introduction of a disulfide bond in the latch resulted in an increase in affinity for peptides corresponding to host ligand, which bind to the cleft in an antiparallel fashion. Interestingly, the converse was observed when disulfide bonds were added to the latch of SdrG (39), which binds the host peptide mimetic in a parallel orientation (21).

Although the biological implications of this difference in affinity are unclear, it is noteworthy that Srr2 is expressed exclusively by GBS serotype III strains belonging to the ST-17 clonal complex. These organisms have been strongly associated with neonatal invasive infections (16, 22–26, 53). Few ST-17-specific virulence factors have been described to explain this enhanced pathogenicity of ST-17 strains, although it is likely that more than one virulence determinant contributes to the hypervirulence of this sequence type (27, 53). Of note, the surface protein HvgA is exclusively expressed by ST-17 strains, and its expression has been shown to promote GBS attachment to endothelial and epithelial cell lines (53). However the host receptor for HvgA remains to be elucidated.

Intriguingly, in contrast to the binding trenches, there is significant sequence homology among the latch-binding subdomain (“latching cleft”) of Srr1 and Srr2 (Table 4), with both having a conserved cleft motif. Alignment of the Srr1- and Srr2-predicted latching clefts with those of other DLL adhesins showed that Srr1 has closer homology to these other DLL proteins, as compared with Srr2. Thus although the structure of the latch may control binding affinity, the influence of sequence differences in the latching clefts on the structure of the latch is unclear.

**TABLE 4:** **Putative latching cleft and latch sequences of DLL proteins**

DLL-binding adhesin	Latching cleft	Cleft-latch residue spacing	Latch motif	Organism	Ref.
ClfA	IYTFTDYVN	207	GSGSGDG	*S. aureus*	43
ClfB	TFVFTDYVN	204	GGGSADG	*S. aureus*	19
SdrC	TYTFTNYVD	196	GSSTANG	*S. aureus*	21
SdrD	TYTFTDYVD	212	NQSGGAG	*S. aureus*	21
SdrE	TYTFTDYVD	216	GGGDGTV	*S. aureus*	21
SdrG	TYTFTDYVD	207	SSGQGQG	*S. epidermidis*	21
SdrF	TYTFTNYVD	200	GSSTAQG	*S. epidermidis*	21
FnbpA	RYTFTNDIE	210	NKANGNE	*S. aureus*	21, 35
FnbpB	RYTFKEYVQ	204	NNAQGDG	*S. aureus*	21, 52
Srr1	TYTWTRYAS	209	GDSDANA	*S. agalactiae*	4
Srr2	VYSFTDFAA	200	GYSDVNA	*S. agalactiae*	This study

The binding site on fibrinogen for staphylococcal adhesins is typically composed of about 20 amino acids on one of the fibrinogen chains. For example, ClfA recognizes the C-terminal 17 residues of the γ chain (GEGQQHHLGGAKQAGDV); ClfB binds to 16 residues (tandem repeat 5; GSWNSGSSGTGSTGNQ) in the αC domain of the Aα chain, and SdrG binds the N-terminal 20 residues of the β chain (NEEGFFSARGHRPLDKKREE) (19, 43, 50). Although Srr1 and Srr2 also interact with the C terminus of the Aα chain, the binding site for both adhesins is contained within the adjacent tandem repeats 6–8 of the protein (NPGSPRPGSTGTWNPGSSERGSAGHWTSESSVSGSTGQW). Thus, not only do these bacterial surface proteins bind fibrinogen via a DLL-like mechanism, but these adhesins can interact with different regions of fibrinogen.

*In vivo*, GBS may interact with fibrinogen through several pathways, in addition to Srr1- or Srr2-mediated binding. Studies by Harris *et al.* (54) identified a cell wall-anchored protease of GBS (CspA) that both cleaved the fibrinogen Aα chain *in vitro* and appeared to mediate fibrinogen-dependent aggregation of whole bacteria. Deletion or disruption of *cspA* was associated with both increased opsonophagocytosis by neutrophils and decreased virulence in an animal model of neonatal sepsis. In addition, GBS may complement binding of Srr1 and Srr2 with binding by other adhesins. FbsA and FbsB are two additional surface proteins that also mediate GBS binding to human fibrinogen (55–59). These adhesins appear to be structurally unrelated to each other or the SRR proteins, and neither protein is associated with a DLL binding mechanism. The binding site on fibrinogen for FbsA is contained within the D fragment (60) but has not been further characterized. No binding site for FbsB has as yet been identified. Expression of FbsA enhances GBS binding to human epithelial and endothelial cells (61, 62), but it does not appear to contribute to cell invasion (63). In addition, fibrinogen binding via FbsA reduced uptake by a macrophage cell line (64), indicating that it may block phagocytosis. Deletion of *fbsA* attenuated the virulence of GBS in animal models of arthritis and septicemia (65), indicating that this protein contributes to pathogenicity of the organism. FbsB promotes GBS invasion of human brain microvascular cells (58), although the *in vivo* relevance of this phenotype remains to be examined. Recent proteomic screening identified two additional fibrinogen-binding proteins expressed by GBS, the fibronectin-binding protein Fib and a predicted ABC transporter (SAG0242) (66). The mechanisms for fibrinogen binding by these proteins are unknown, and the importance of these interactions for colonization or virulence has not as yet been described.

The results presented in this study show that Srr2 is an ST-17-specific surface protein that, like Srr1 (present in other GBS sequence types), interacts with fibrinogen through a DLL mechanism and promotes GBS attachment to human brain endothelial cells. Previous *in vivo* studies have shown that strains expressing Srr2 are more virulent than Srr1-expressing strains, as measured by a mouse model of sepsis (16). These findings suggest that, although both Srr proteins interact with fibrinogen, the increased affinity of Srr2 for the protein may be one factor contributing to the enhanced pathogenicity and invasive disease associated with ST-17 strains. Studies to address these issues are now in progress.

## Footnotes

2 The abbreviations used are:

SRR: serine-rich repeat
GBS: group B streptococci (*Streptococcus agalactiae*)
DLL: dock, lock, and latch
ITC: isothermal calorimetry
hBMEC: human brain microvascular endothelial cells
MSCRAMM: microbial surface components recognizing adhesive matrix molecules
CFU: colony-forming unit
MBP: maltose-binding protein
SPR: surface plasmon resonance
BR: binding region
RU: repeating unit
LS-CAT: Life Sciences Collaborative Access Team.
